# Transcriptome analysis of *Aspergillus niger xlnR* and *xkiA* mutants grown on corn Stover and soybean hulls reveals a highly complex regulatory network

**DOI:** 10.1186/s12864-019-6235-7

**Published:** 2019-11-14

**Authors:** Claire Khosravi, Joanna E. Kowalczyk, Tania Chroumpi, Evy Battaglia, Maria-Victoria Aguilar Pontes, Mao Peng, Ad Wiebenga, Vivian Ng, Anna Lipzen, Guifen He, Diane Bauer, Igor V. Grigoriev, Ronald P. de Vries

**Affiliations:** 1 Fungal Physiology, Westerdijk Fungal Biodiversity Institute & Fungal Molecular Physiology, Utrecht University, Utrecht, the Netherlands; 2 US Department of Energy Joint Genome Institute, Walnut Creek, CA USA; 3 Department of Plant and Microbial Biology, University of California, Berkeley, CA USA

**Keywords:** Transcriptomics, *Aspergillus Niger*, XlnR, XkiA, Gene expression

## Abstract

**Background:**

Enzymatic plant biomass degradation by fungi is a highly complex process and one of the leading challenges in developing a biobased economy. Some industrial fungi (e.g. *Aspergillus niger*) have a long history of use with respect to plant biomass degradation and for that reason have become ‘model’ species for this topic. *A. niger* is a major industrial enzyme producer that has a broad ability to degrade plant based polysaccharides. *A. niger* wild-type, the (hemi-)cellulolytic regulator (*xlnR*) and xylulokinase (*xkiA1*) mutant strains were grown on a monocot (corn stover, CS) and dicot (soybean hulls, SBH) substrate. The *xkiA1* mutant is unable to utilize the pentoses D-xylose and L-arabinose and the polysaccharide xylan, and was previously shown to accumulate inducers for the (hemi-)cellulolytic transcriptional activator XlnR and the arabinanolytic transcriptional activator AraR in the presence of pentoses, resulting in overexpression of their target genes. The *xlnR* mutant has reduced growth on xylan and down-regulation of its target genes. The mutants therefore have a similar phenotype on xylan, but an opposite transcriptional effect. D-xylose and L-arabinose are the most abundant monosaccharides after D-glucose in nearly all plant-derived biomass materials. In this study we evaluated the effect of the *xlnR* and *xkiA1* mutation during growth on two pentose-rich substrates by transcriptome analysis.

**Results:**

Particular attention was given to CAZymes, metabolic pathways and transcription factors related to the plant biomass degradation. Genes coding for the main enzymes involved in plant biomass degradation were down-regulated at the beginning of the growth on CS and SBH. However, at a later time point, significant differences were found in the expression profiles of both mutants on CS compared to SBH.

**Conclusion:**

This study demonstrates the high complexity of the plant biomass degradation process by fungi, by showing that mutant strains with fairly straightforward phenotypes on pure mono- and polysaccharides, have much less clear-cut phenotypes and transcriptomes on crude plant biomass.

## Background

*Aspergillus niger* is a filamentous fungus that degrades plant biomass polysaccharides, such as cellulose, hemicellulose and pectin into monomeric sugars that can serve as a carbon source. Cellulose has a simple structure as a linear polymer of D-glucose. Hemicelluloses are more complex heterosaccharides with many variations in their structure. Pectins are a family of complex polysaccharides with D-galacturonic acid as the main monomeric component. The composition of plant biomass is detailed in Table 1. *A. niger* is able to secrete a broad spectrum of enzymes that can hydrolyze polysaccharides into pentoses, hexoses and other monomeric components [1], which can be taken up by the fungus. *A. niger* then uses a variety of catabolic pathways to efficiently convert the monomeric components of plant biomass. Significant progress has been made in the utilization and conversion of cellulose-derived hexose sugars into bioethanol. Several reports summarized the latest developments from 1st generation to 2nd generation (2G) ethanol technologies [2]. However, the use of pentose sugars, such as L-arabinose and D-xylose presents an opportunity to increase the efficiency of 2G bioethanol. In *A. niger* the release of L-arabinose and D-xylose from plant biomass requires the synergistic action of several Carbohydrate Active enZymes (CAZymes) [1]. After release from the polymers, L-arabinose and D-xylose are metabolized through the pentose catabolic pathway (PCP), consisting of oxidation, reduction and phosphorylation reactions to form D-xylulose-5-phosphate, which enters the pentose phosphate pathway (PPP) [3–5]. The PPP is one of the central metabolic pathways in primary carbon metabolism. The production of D-xylulose-5-phosphate from the PCP enables the fungus to answer efficiently to the increased demands of NADH and NADPH [6].

**Table 1:** Composition of plant biomass. Based on Kowalczyk et al.*,* 2014

Biomass	Polymer	Monomers
Cellulose		D-glucose
Hemicellulose	Xylan	D-xylose
	Glucuronoxylan	D-glucuronic acid, D-xylose
	Arabinoglucuronoxylan	D-xylose, L-arabinose
	Arabinoxylan	D-xylose, L-arabinose
	Galacto(gluco)mannan	D-glucose, D-mannose, D-galactose
	Mannan/galactomannan	D-mannose, D-galactose
	Xyloglucan	D-glucose, D-xylose, D-fructose, D-galactose
	β(1,3)/(1,4)-Glucan	D-glucose
Pectin	Homogalacturonan	D-galacturonic acid
	Xylogalacturonan	D-galacturonic acid, D-xylose
	Rhamnogalacturonan I	D-galacturonic acid, L-rhamnose, D-galactose, L-arabinose, ferulic acid, D-glucuronic acid
	Rhamnogalacturonan II	D-galacturonic acid, L-rhamnose, D-galactose, L-arabinose, L-fucose, D-glucose, D-manno-octulosonic acid (KDO), D-lyxo-heptulosaric acid (DhA), D-xylose, D-apiose, L-acetic acid
Inulin		D-fructose, D-glucose
Starch	Amylose	D-glucose
	Amylopectin	D-glucose
Various gums		D-galacturonic acid, L-rhamnose, D-galactose, L-arabinose, D-xylose, L-fucose (depending on the specific gum type)
Lignin		monolignols: ρ-coumaryl alcohol, coniferyl alcohol, sinapyl alcohol

In *A. niger*, the xylanolytic enzyme system is regulated by the zinc binuclear transcription factor (TF) XlnR [5, 7–12]. In addition to extracellular enzymes, XlnR also regulates D-xylose reductase (*xyrA*) in the PCP, and ribose-5-isomerase (*rpiA*) and transaldolase (*talB*) in the PPP [13]. Activation of XlnR depends on the presence of D-xylose that acts as an inducer, released from the environment by low level constitutively expressed or starvation-influenced scouting enzymes [13–17]. It has been demonstrated that D-xylose induction is concentration-dependent: acting as an inducer for xylanases at low concentrations and as a repressor through CreA at higher concentrations [14, 18]. Another TF, AraR, has been identified in *A. niger* and was shown to interact with XlnR in the regulation of the PCP [5, 13].

Corn stover (CS) and soybean hulls (SBH) are commonly used as renewable feedstocks for many applications. CS has strong advantages as a feedstock for energy, chemicals, and materials, because of its high volume and low cost [19]. CS contains stalks, leaves, tassel, husk, and cob from the corn crop [20], making it highly heterogeneous. The composition of each fraction varies, and each fraction is known to respond differently to enzymatic hydrolysis [21–23]. Crude CS consists of 37.1% cellulose, 20.9% hemicellulose, 13.5% lignin, and 1.3% ash [24].

Soybean hulls (SBH) is the predominant by-product from the soybean process industry [25]. The chemical composition of SBH may contain variable amounts of cellulose (29–51%), hemicellulose (10–25%), lignin (1–4%), pectin (4–8%), proteins (11–15%), and minor extractives [25]. Lignin is the most recalcitrant component of the plant cell wall. SBH is easy degradable due to its low level of lignin and is therefore attractive as a potential feedstock for fuel and other industrial uses.

Different pretreatment methods have been studied in relation to the production of monomeric sugars from CS and SBH [21, 26]. However, the costs of cellulase and hemicellulase production contribute significantly to the price of biofuel. Improving the methods to obtain these enzyme cocktails and increasing their efficiency is a key factor to make biofuels economically sustainable. One of the possibilities to optimize the biofuel production process is the genetic engineering of enzyme production organisms, such as *A. niger.*

The role of XlnR in regulation of enzyme production was studied in detail on monosaccharides and polysaccharides, but the role of this TF on two natural substrates like CS and SBH has been studied less extensively. In this study we describe a transcriptomic analysis of *A. niger* wild-type, Δ*xlnR* and *xkiA1* mutant grown on CS and SBH. The goal was to analyze the effect of the deletion of *xlnR* and *xkiA1* over time during growth on these substrates. Our hypothesis in this study was that at an early time point the XlnR target genes would have reduced expression in Δ*xlnR* and are up-regulated in *xkiA1* mutant due to accumulation of the inducers of XlnR and AraR. Previous studies demonstrated that transcript levels of several genes encoding cellulolytic, xylanolytic and xyloglucanolytic enzymes were decreased in an *xlnR* deletion mutant [10, 27, 28]. In contrast, increased transcript levels of genes encoding arabinan and xylan degrading enzymes have been observed in the *xkiA1* mutant, as well as intracellular accumulation of L-arabitol and xylitol [3, 5, 29]. At the later time points of our study, we expected *A. niger* to compensate for these mutations by using other regulatory mechanisms. Interestingly, our results demonstrated that the response of *A. niger* to crude plant biomass substrates is even more complex than could be extrapolated from studies on pure mono- and polysaccharides.

## Results and discussion

### Growth profile of *A. niger* wild-type, *xkiA1* and Δ*xlnR*

**Fig. 1:**
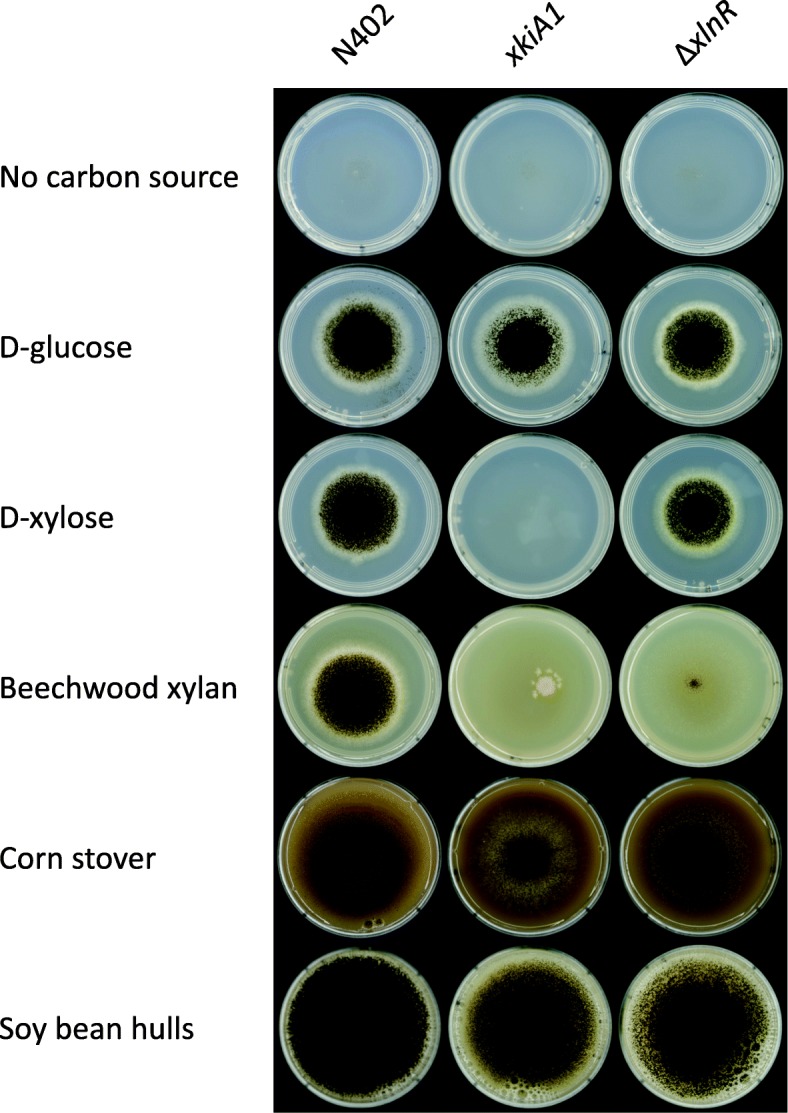
Growth of *Aspergillus niger* wild-type N402, *xkiA1* and Δ*xlnR* strains on no carbon source, 25 mM D-glucose, 25 mM D-xylose, 1% beechwood xylan, 3% corn stover and 3% soybean hulls, after 3 days of growth at 30 degrees

The three strains were grown on minimal medium containing no carbon source, 25 mM D-glucose, 25 mM D-xylose, 1% beechwood xylan, 3% corn stover or 3% soy bean hulls (Fig. 1). As has been shown before, the *xkiA1* mutant was not able to grow on D-xylose (due to a block in the pentose catabolic pathway [30]) and had only residual growth on beechwood xylan (due to other sugars than D-xylose in this substrate), while the *xlnR* deletion strain had only a small reduction in growth on D-xylose (due to compensation of AraR [5, 31]) and strongly reduced growth on beechwood xylan (due to reduced expression of xylanases [10]).

Interestingly, on corn stover and soy bean hulls, both strains had a very similar phenotype, which was somewhat less growth than the wild type. This indicates that during growth on crude plant biomass, the influence of these mutations is significantly smaller than on xylan, most likely due to the presence of other polymers that can serve as alternative carbon sources. The net burden of either blocking pentose catabolism or significantly reduced production of xylanolytic genes can apparently be compensated for by other systems. Therefore, we studied the response of these strains in detail by using transcriptomics.

### Overall effect of *xlnR* and *xkiA1* deletion on the CAZy genes involved in the plant biomass degradation

To gain more insight into the regulation of cellulose-, hemicellulose- and pectin-degrading enzymes by XlnR on a natural substrate, the wild-type strain and the mutant strains Δ*xlnR* and *xkiA1* were pre-grown in liquid cultures containing MM with D-fructose, and then transferred to MM with 1% CS or 1% SBH for 4, 24 and 48 h. RNA-seq analysis was performed and the transcriptome response during growth on CS and SBH was analyzed in the mutants compared to the wild-type strain. On average 98% of the reads were mapped to the genome and 80% of the reads were mapped to a gene. Based on previous studies on monosaccharides and polysaccharides, it was expected that XlnR-target genes will be reduced in expression in the *xlnR* mutant and up-regulated in the *xkiA1* mutant at the early time point [29]. The expression data were analyzed to evaluate whether this is also the case on a crude substrate consisting of multiple monomeric compounds. *A. niger* XlnR is involved in degradation of cellulose, xylan, xyloglucan and to some extent galactomannan [9–11, 32]. The *xkiA1* mutant is an UV mutant, unable to grow on L-arabinose and D-xylose and deficient in D-xylulose kinase activity [3, 29]. XkiA is essential for the utilization of D-xylose and L-arabinose, which are major components of xylan, xyloglucan and pectin. Since CS contains mainly cellulose and xylan, and SBH mainly cellulose, xyloglucan and pectin, we evaluated the effects of the deletion of *xlnR* and *xkiA1* on CAZy genes related to these polysaccharides. Principle Component Analysis was performed on the transcriptome data to verify the reproducibility of the biological replicates (Additional file 1: Figure S1). This also demonstrated that the pre-cultures of the *xlnR* deletion strain differed from those of the other strains. While we did not see strong overlap in the set of differentially expressed genes of the pre-culture and the later samples, we cannot fully exclude that this difference in the pre-culture may have some effect on the expression of the later samples.

**Fig. 2:**
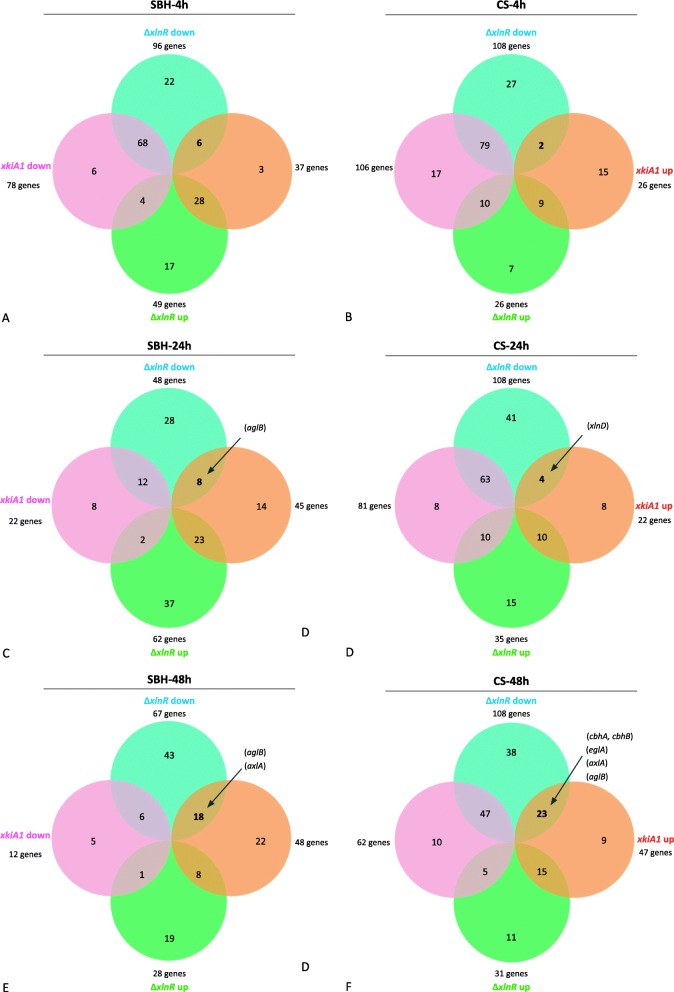
Venn diagrams showing the CAZy genes involved in the degradation of plant biomass in *A. niger* that are significantly up-regulated and down- regulated genes in SBH (**a**, **c**, **e**) and CS (**b**, **d**, **f**) between Δ*xlnR* vs the wild-type (green and blue) and between *xkiA1* vs the wild-type (orange and pink) after 4 h (**a**; **b**), 24 h (**c**; **d**) and 48 h (**e**, **f**). The gene numbers are listed in Additional file 3: Table S1

**Fig. 3:**
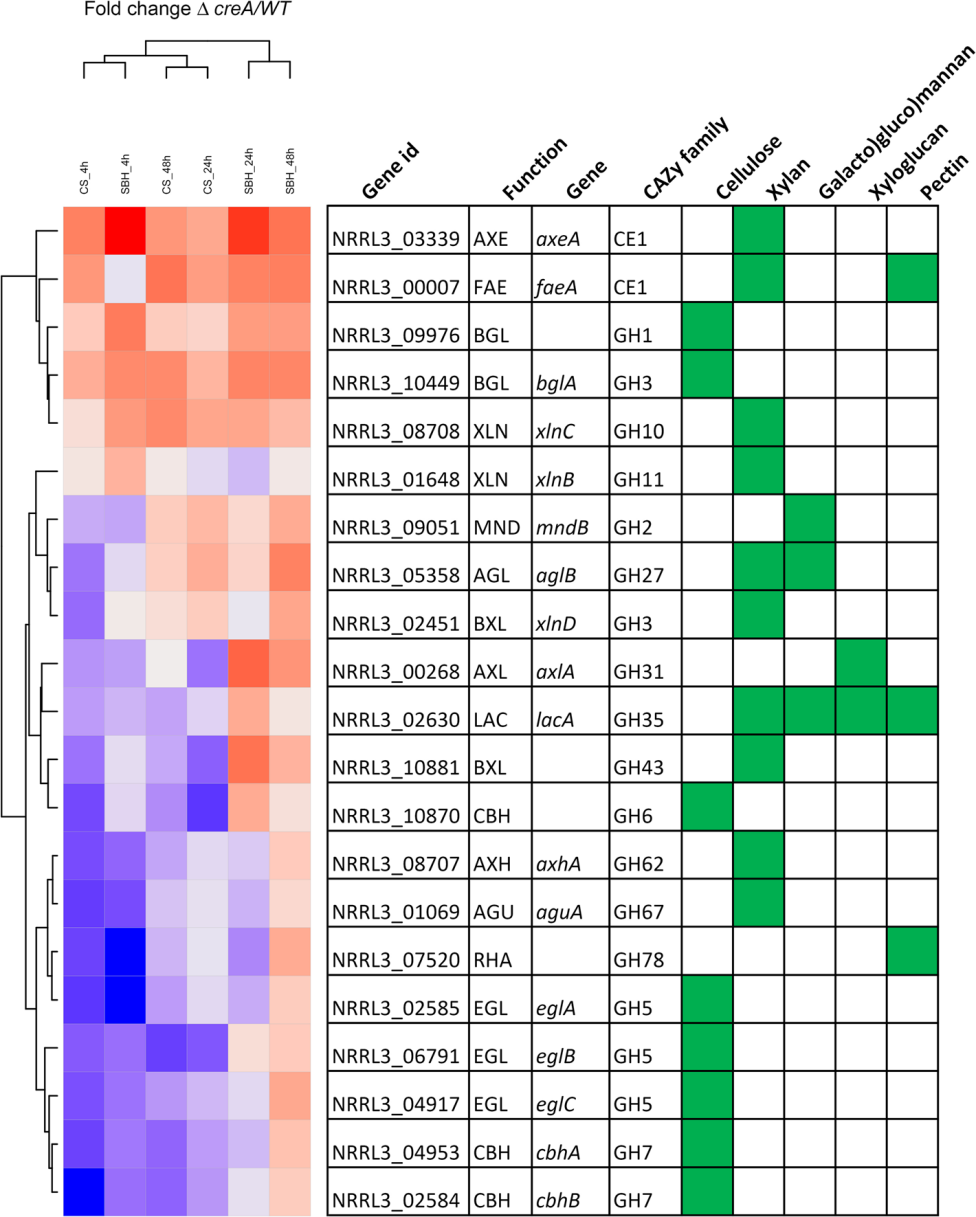
Hierarchical clustering of expression of genes regulated by XlnR in the *A. niger* Δ*xlnR* mutant compared to the wild-type after 4 h, 24 h, 48 h of transfer to 1% corn stover (CS) or 1% soybean hulls (SBH). The polysaccharide the genes are related to are indicated in green

Genes were considered differentially expressed if the log2 fold change was greater than 0.6 or less than − 0.6 with adjusted *p*-value ≤0.05. GO-term enrichment demonstrated that in particular genes related to carbohydrate metabolism were affected in the strains (Additional file 2: Figure S2; Additional file 3: Table S1), so we focused on these gene groups in our study. The difference in CAZy gene expression of Δ*xlnR* and the *xkiA1* mutant compared to the wild-type was analyzed over time (4, 24 and 48 h). After 4 h on CS 108 genes had reduced expression in Δ*xlnR* and from those genes, two were up-regulated and 79 were down-regulated in the *xkiA1* mutant (Fig. 2; Additional file 4: Table S2). Similar results were observed after 24 h on CS, with 108 genes that were down-regulated in Δ*xlnR* of which four were up-regulated and 63 were down-regulated in the *xkiA1* mutant. After 48 h on CS 108 genes were down-regulated in Δ*xlnR* and from them 23 were up-regulated and 47 were down-regulated in the *xkiA1* mutant, indicating that the highest number of CAZy genes showed the expected profile of down-regulated in the *xlnR* mutant and up-regulated in the *xkiA1* mutant at the latest time point. Expression of a previously identified set of 21 XlnR-dependent targets genes was evaluated in our data-set (Fig. 3), most of which were significantly down-regulated in Δ*xlnR*. The exception was an α-rhamnosidase encoding gene (NRRL3_07520) after 4 h of transfer to CS. Interestingly, after 24 h of transfer to CS, of the four genes down-regulated in Δ*xlnR* and up-regulated in the *xkiA1* mutant, only one gene has been identified as an XlnR-target gene: β-xylosidase (BXL; *xlnD*) (Fig. 3). After 48 h of transfer to CS, of the 23 genes that were down-regulated in Δ*xlnR* and up-regulated in the *xkiA1* mutant, two genes have been previously identified as XlnR-target genes: an α-galactosidase (AGL; *aglB*) and an α-xylosidase (AXL; *axlA*). Overall, the set of genes responding to the mutations differs from those observed on xylan or D-xylose, indicating the more complex regulatory system that is active during growth on crude plant biomass.

After 4 h on SBH, 96 genes were down-regulated in Δ*xlnR* and of those genes six were up-regulated and 68 were down-regulated in the *xkiA1* mutant (Fig. 2; Additional file 4: Table S2). Compared to CS, there was a larger shift in the expression profiles between the time points, since after 24 h on SBH, only 48 genes were down-regulated in the Δ*xlnR* strain of which eight were up-regulated and 12 were down-regulated in the *xkiA1* mutant. After 48 h on SBH 67 genes were down-regulated in Δ*xlnR*. From these, 18 were up-regulated and six were down-regulated in the *xkiA1* mutant. As was observed for CS, after 48 h the highest number of CAZy genes showed the expected profile of being down-regulated in the *xlnR* deletion mutant and up-regulated in the *xkiA1* mutant. One α-galactosidase (AGL; *aglB*), two cellobiohydrolases (CBH; *cbhA* and *cbhB*) and one endoglucanase (EGL; *eglA*) were down-regulated in Δ*xlnR* and up-regulated in the *xkiA1* mutant after 24 h and 48 h of transfer to SBH. In addition, *axlA* was down-regulated in Δ*xlnR* and up-regulated in the *xkiA1* mutant after 48 h of transfer to SBH (Fig. 2; Additional file 4: Table S2).

Overall, larger differences were observed in SBH compared to CS after 24 h and 48 h. A higher number of CAZy genes were up-regulated in the *xkiA1* mutant, especially pectinases, on SBH compared to CS after 24 h. Our results showed an antagonistic effect between Δ*xlnR* and the *xkiA1* mutant after 48 h to CS and SBH, since more genes were up-regulated in the *xkiA1* mutant compared to Δ*xlnR*, while more genes were down-regulated in Δ*xlnR* compared to the *xkiA1* mutant*.*

### Expression of cellulolytic genes

**Fig. 4:**
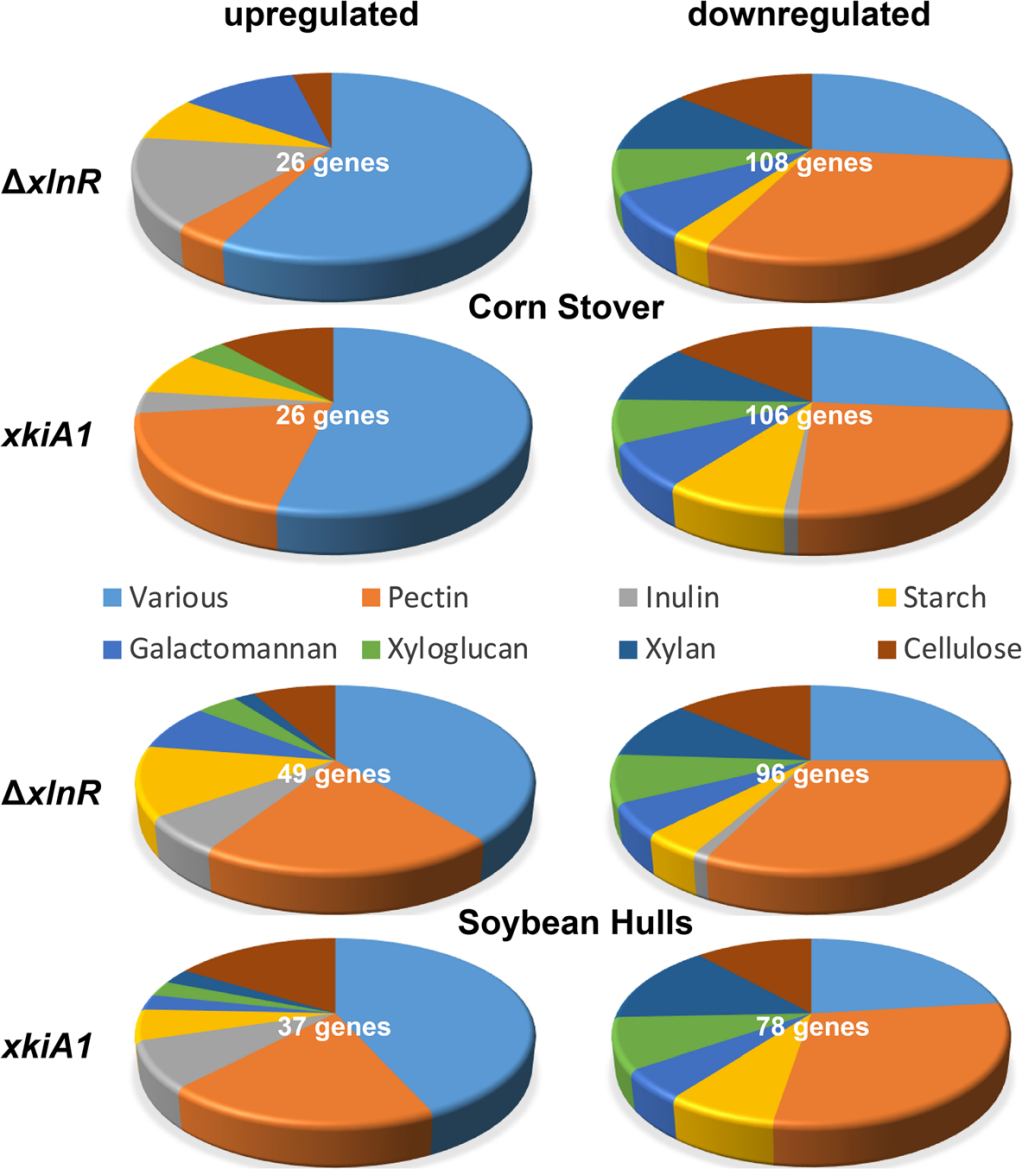
Pie-chart presenting the proportion of CAZy genes involved in the degradation of different plant polysaccharides in *A. niger* that are significantly up-regulated or down-regulated between Δ*xlnR* vs the wild-type and between *xkiA1* vs the wild-type after 4 h of transfer to Corn Stover and Soybean Hulls. The gene numbers are listed in Additional file 3: Table S1

**Fig. 5:**
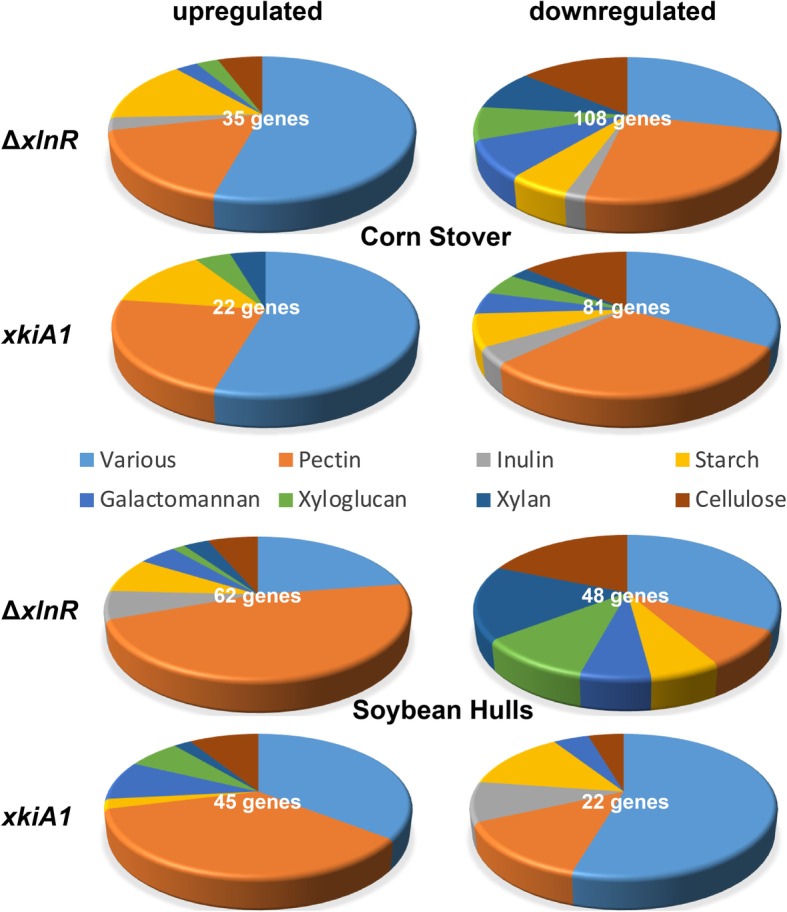
Pie-chart presenting the proportion of CAZy genes involved in the degradation of different plant polysaccharides in *A. niger* that are significantly up-regulated or down-regulated between Δ*xlnR* vs the wild-type and between *xkiA1* vs the wild-type after 24 h of transfer to Corn Stover and Soybean Hulls. The gene numbers are listed in Additional file 3: Table S1

**Fig. 6:**
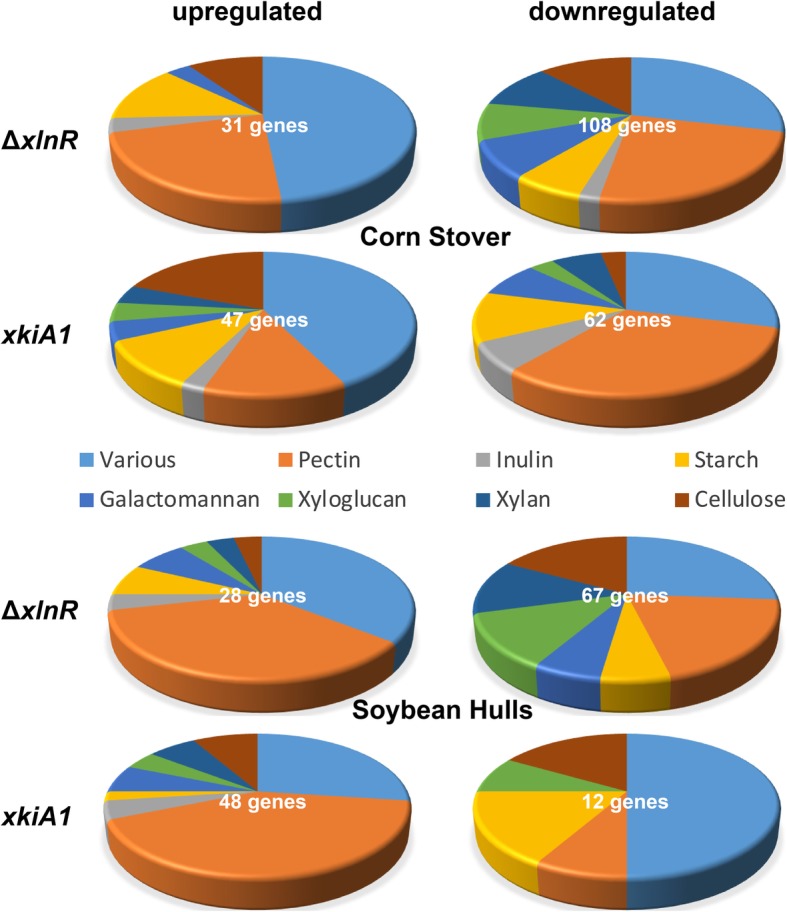
Pie-chart presenting the proportion of CAZy genes involved in the degradation of different plant polysaccharides in *A. niger* that are significantly up-regulated or down-regulated between Δ*xlnR* vs the wild-type and between *xkiA1* vs the wild-type after 48 h of transfer to Corn Stover and Soybean Hulls. The gene numbers are listed in Additional file 3: Table S1

After 4 h and 24 h of transfer to CS, 15 cellulolytic CAZy genes were down-regulated in Δ*xlnR* compared to the wild-type, while after 48 h, 13 cellulolytic CAZy genes were down-regulated (Figs. 4, 5 and 6; Additional file 4: Table S2, Additional file 5: Figure S3). Some cellulolytic genes were up-regulated in the Δ*xlnR* strain at all three tested time-points. In the *xkiA1* mutant after 4 h and 24 h a similar trend can be observed; most cellulolytic genes were down-regulated and only a few genes were up-regulated, but after 48 h the opposite effect was observed. Two cellulolytic genes were down-regulated and ten were up-regulated in the *xkiA1* mutant compared to the wild-type*.*

In SBH, the same trend as for CS was observed in Δ*xlnR*, in that the majority of cellulolytic genes were down-regulated at all the time points tested (Figs. 4, 5 and 6; Additional file 4: Table S2, Additional file 5: Figure S3), but a lower number of genes were differentially expressed in the *xkiA1* mutant compared to CS*.* Several cellulolytic genes, previously identified as XlnR-target genes showed interesting transcript profiles. Two endoglucanases (EGL; *eglA* and *eglC*) [10, 32] were down-regulated at all time points in both substrates, while a third EGL, *eglB*, was only down-regulated after 24 h in CS and after 4 h in SBH. Two XlnR-regulated cellobiohydrolases (CBH; *cbhA* and *cbhB*) [11] were down-regulated at all the time points in CS, while in SBH, *cbhA* was down-regulated only after 4 h and *cbhB* after 4 h and 48 h. Interestingly, *eglA*, *cbhA* and *cbhB* showed the expected profile, down-regulated in Δ*xlnR* and up-regulated in the *xkiA1* mutant, but only after 48 h of transfer to CS and not at the earlier time points.

### Expression of xylan and xyloglucan genes

At all time points tested in CS and SBH, the majority of the xylanolytic genes and xyloglucan-specific genes were down-regulated in Δ*xlnR*. After 4 h in CS most of the xylanolytic genes and xyloglucan-specific genes were also down-regulated in the *xkiA1* mutant, but after 24 h, the effect of the *xkiA1* mutation is less pronounced, and after 48 h more xyloglucan- specific genes were up-regulated, compared to the earlier time points (Figs. 4, 5 and 6; Additional file 4: Table S2, Additional file 5: Figure S3)*.*

No major differences were observed after 4 h in SBH in the *xkiA1* mutant compared to Δ*xlnR.* After 24 h, unlike in CS, no xylanolytic genes and xyloglucan-specific genes were down-regulated in SBH in the *xkiA1* mutant. After 48 h no xylanolytic genes were down-regulated in SBH in the *xkiA1* mutant compared to the wild-type, whereas four were down-regulated in CS*.* Previously, two endoxylanases (XLN; *xlnA*, *xlnB*) and a β-xylosidase (BXL, *xlnD*) have been identified as XlnR-target genes [9, 10]. In our RNA-seq analysis, *xlnA* and *xlnB* were down-regulated at all time points in both substrates, while *xlnD* was also down-regulated at all-time point in CS, but only after 4 h and 24 h in SBH. These genes were in general not up-regulated in the *xkiA1* mutant, with the exception that *xlnD* was up-regulated only after 24 h on CS.

### Expression of pectinolytic genes

At all the time points tested, most of the pectinolytic genes were down-regulated in CS in both Δ*xlnR* and the *xkiA1* mutant (Figs. 4, 5 and 6; Additional file 4: Table S2, Additional file 5: Figure S3). In contrast, after 4 h in SBH, ten pectinolytic genes were up-regulated, while only one was up-regulated in CS in Δ*xlnR*. This became even more pronounced after 24 h, when twenty-nine pectinolytic genes were up-regulated in SBH, whereas only six were up-regulated in CS in Δ*xlnR*. In contrast, only four were down-regulated at this time point in SBH. Interestingly, this pattern changed after 48 h, as then thirteen pectinolytic genes were down-regulated in SBH, and twenty-six were down-regulated in CS in Δ*xlnR*, and the number of up-regulated genes reduced to ten for SBH and seven for CS*.*

The pectinolytic expression profiles of the *xkiA1* mutant in CS and SBH after 24 h were similar to Δ*xlnR,* with sixteen pectinolytic genes that were up-regulated in SBH, while only five were up-regulated in CS. However, unlike for Δ*xlnR,* this effect was still observed after 48 h.

Overall, pectinolytic gene expression seems to go up in the absence of XlnR and to a smaller extent XkiA on SBH, which could be explained by the use of L-rhamnose and/or D-galacturonic acid as an alternative carbon source, which is highly present in this substrate. This would be expected to result in increased induction of GaaR and RhaR, two of the main activators of pectinolytic genes, but this was not observed (see below). Alternatively, these regulators may be under post-transcriptional control in the presence of these compounds, as was shown for XlnR on D-xylose [33].

### Expression of CAZy genes related to other plant biomass components

The expression of CAZy genes related to other plant biomass components (galactomannan, starch, and inulin) was also evaluated to determine whether expression of these genes was affected in the mutants. At all time points in CS most of the galactomannan-specific genes, starch-specific genes and CAZy genes acting on various substrates were down-regulated in Δ*xlnR* (Figs. 4, 5 and 6; Additional file 4: Table S2, Additional file 5: Figure S3). One galactomannan-specific gene, previously identified as XlnR-target genes, *aglB* [8], was up-regulated in SBH and down-regulated in CS. However, after 4 h, four inulin-specific genes were up-regulated, while one was down-regulated in CS in Δ*xlnR.* Most of the galactomannan-specific genes and starch-specific genes were down-regulated in the *xkiA1* mutant in CS at all time points, but this was only the case at 4 and 24 h in CS for the CAZy genes acting on various substrates.

After 4 h in SBH, more starch-specific genes were up-regulated in Δ*xlnR* compared to CS. After 24 h three galactomannan-specific genes and four inulin-specific genes were up-regulated in SBH, whereas only one of each group was up-regulated in CS. No major differences were observed after 48 h between CS and SBH in Δ*xlnR.* In the *xkiA1* mutant, after 4 h in SBH, more galactomannan-specific genes, inulin-specific genes and CAZy genes acting on various substrates were up-regulated compared to CS. This effect became less pronounced after 24 and 48 h.

These results showed that the effect of *xlnR* deletion and *xkiA1* mutation on CAZy gene expression changes over time and depends on the composition of the crude substrates. Overall, many CAZy genes involved in the degradation of cellulose, xylan and xyloglucan were down-regulated at all time points tested on both substrates in Δ*xlnR* (Figs. 4, 5 and 6; Additional file 4: Table S2, Additional file 5: Figure S3). In the *xkiA1* mutant most of the cellulolytic, xylanolytic and xyloglucan-specific genes were down-regulated after 4 h in both substrates as observed for Δ*xlnR*. After 4 h in CS or SBH, *xlnR* and *xkiA1* mutants respond in a similar way, suggesting that at this early time point inability to use pentoses is the main effect on the expression profiles rather than the difference in the mutation causing this.

However, after 24 h and 48 h differences were observed in both deletion mutants between the two crude substrates. In the *xkiA1* mutant, a higher number of cellulolytic genes were down-regulated after 24 h and up-regulated after 48 h in CS, compared to SBH. Also, after 24 h, more xylanolytic and xyloglucan-specific genes were down-regulated in SBH compared to CS in the *xkiA1* mutant*.* After 24 h in SBH a high number of enzymes acting on the different substructures of the pectin, homogalacturonic acid (HGA), rhamnogalacturonan I (RG-I) and side chains (SC) were up-regulated in both mutants compared to CS.

After 48 h, a high number of pectinases were up-regulated in SBH in the *xkiA1* mutant. Our data showed that the mutation of *xkiA1* results in up-regulation, whereas the deletion of *xlnR* results in down-regulation of several CAZymes involved in plant biomass degradation. This demonstrates that a metabolic and regulatory mutation with the same phenotype when grown on pure monosaccharides can result in a different physiology during prolonged growth on crude substrates.

Previously, we demonstrated the dominant role of XlnR in colonization and degradation of wheat bran [34]. During the late colonization stage (40 h post inoculation), only the strains in which *xlnR* was deleted were unable to colonize the smooth surface of wheat bran, due to the absence/reduction of several cellulolytic and arabinoxylanolytic enzymes. These results correlate with the down-regulation of CAZymes involved in the degradation of cellulose, xylan, xyloglucan and galactomannan observed in the Δ*xlnR* strain on CS and SBH. The expression profiles of seven selected genes were confirmed by qRT-PCR, to validate the RNAseq data (Additional file 6: Figure S4).

### Expression profiles of other regulators involved in the degradation of CS and SBH and their metabolic target genes

The monomeric composition of CS and SBH is detailed in Table 2. CS and SBH contain various polysaccharides and provide options for consumption of other sugars than hexoses, for example pentoses (D-xylose and L-arabinose) and uronic acids. It is important to notice that the uronic acid level is higher in SBH than in CS and it also consists of different quantities of the other monomeric sugars. In SBH, the uronic acid fraction consists mainly of D-galacturonic-acid, while CS contains (4-(O)-methyl-)D-glucuronic-acid [1]. These differences in composition not only imply variation in the presence or levels of inducers for plant biomass related transcriptional regulators, but also the need –to activate different metabolic pathways in time to optimally use the two substrates.

**Table 2:** Composition of the substrates used in this study

Mol%	L-rhamnose	D-fucose	L-arabinose	D-xylose	D-mannose	D-galactose	D-glucose	Uronic acid	Total
SBH	1.0	0.0	8.4	15.0	7.1	4.0	50.0	15.9	68.0
CS	0.4	0.0	4.6	34.9	0.7	1.7	53.4	4.3	59.5

To analyze the effect of *xlnR* or *xkiA1* mutants on sugar catabolism, expression of genes involved in conversion of L-arabinose/D-xylose, L-rhamnose, and D-galacturonic acid, and the regulators controlling them, was analyzed in the *xlnR* and *xkiA1* mutants compared to the wild-type strain grown on CS and SBH for 4 h, 24 h, and 48 h. Expression of other TFs involved in the cellulose, hemicellulose and pectin degradation was also analyzed to determine the effect of *xlnR* or *xkiA1* mutants on their expression.

### The L-arabinose-responsive regulator AraR

AraR regulates most genes involved in the PCP: L-arabinose reductase (*larA*), L-arabitol dehydrogenase (*ladA*), L-xylulose reductase (*lxrA*), xylitol dehydrogenase (*xdhA*) and D-xylulokinase (*xkiA1*) during growth on L-arabinose *in A. niger* [5, 13]. The later two genes as well as D-xylose reductase (*xyrA*) are under control of XlnR during growth on D-xylose. In addition, *rpiA* and *talB* have been identified as XlnR regulated genes. After 4 h of transfer to CS or SBH all the genes involved in the PCP were down-regulated in Δ*xlnR*, but only on SBH in the *xkiA1* mutant compared to the wild type strain (Additional file 4: Table S2, Additional file 7: Table S3). Interestingly, after 24 h and 48 h, the previously identified XlnR-target genes from the PCP, *xyrA* and *xdhA,* were down-regulated in both substrates at all time-points tested in Δ*xlnR*. XlnR seems to have a major influence on the expression of *xdhA* on both CS and SBH because in absence of XlnR, we do not observe the antagonistic interaction of AraR in regulation of this gene. None of the other PCP genes (*larA*, *ladA*, *lxrA* and *xkiA*) were consistently down-regulated in Δ*xlnR* (Additional file 7: Table S3, Additional file 8: Figure S5), but sometimes up-regulated at certain late time-points on CS or SBH, which implies that these genes are dependant on both XlnR and AraR on these crude substrates, but with a dominant regulatory role for AraR for the genes involved in the first three steps of te L-arabinose pathway. In Δ*xlnR araR* was up-regulated after 4 and 24 h of transfer to CS or SBH, compared to the wild-type strain (Additional file 9: Table S4). These results correlate well with the previously reported antagonistic interaction of these regulators in *A. niger*, where it was shown that deletion of *xlnR* results in up-regulation of the PCP genes under control of AraR [5].

In the *xkiA1* mutant *araR* was up-regulated after 24 h and 48 h of transfer to CS, but only after 4 h of transfer to SBH (Additional file 9: Table S4). L-arabitol is the inducer from AraR and accumulates in the *xkiA1* mutant during growth on D-xylose or L-arabinose [29]. After 4 h on both CS and SBH *xyrA* and *xdhA* were down-regulated, and similar results were observed for the extracellular enzymes releasing D-xylose residues. After 24 and 48 h, transcript levels of these genes were not consistently down-regulated as observed in the *xlnR* deletion mutant. In contrast, transcript levels of *larA*, *ladA*, *lxrA* and *xkiA* on CS were up-regulated at some of the time points, while this was observed only for *ladA* and *lxrA* on SBH (Additional file 7: Table S3, Additional file 8: Figure S5).

The results in our study indicate that conversion of pentoses and subsequent accumulation of L-arabitol and D-xylose in the *xkiA1* mutant might occur earlier in SBH than in CS. L-arabitol and D-xylose accumulation would cause up-regulation of the XlnR regulated genes at the early time-point on CS and SBH according to our hypothesis. However, the transcript levels of genes involved in the PCP and especially the extracellular response (xylanolytic and xyloglucan-active enzymes) appears to be similar to the *xlnR* deletion mutant after 4 h. We hypothesize that L-arabitol or D-xylose might not have accumulated to a high enough level that it can (hyper) induce the XlnR regulated genes as it has been observed previously during growth D-xylose and L-arabinose [13, 29].

As the PCP and PPP are interconnected, we also evaluated expression of genes involved in the PPP. Several genes involved in the PPP were down-regulated in Δ*xlnR*, after transfer to CS or SBH (Additional file 7: Table S3). As expected, *talB*, previously identified as XlnR regulated gene, was down-regulated in both substrates at all the time points tested [35]. However, the other suggested XlnR-regulated gene (*rpiA*) was only down-regulated after 24 h of transfer to SBH and therefore our results do not conclusively confirm that *rpiA* is only regulated by XlnR. Similarly, none of the other PPP genes were consistently down-regulated at all time points in Δ*xlnR*, which implies that they are not directly regulated by XlnR, but more likely indirectly affected to a different extent at the various time points.

### L-rhamnose responsive regulator (RhaR)

RhaR controls expression of genes involved in RG-I degradation, as well as the L-rhamnose catabolic genes L-rhamnose-1-dehydrogenase (*lraA*), L-rhamnono-γ-lactonase (*lraB*) and L-rhamnonate dehydratase (*lraC*) during growth on L-rhamnose in *A. niger* [36–38]. Interestingly, *rhaR* was up-regulated at all the time points tested in SBH in Δ*xlnR*, as were *lraA*, *lraB* and *lraC* (Additional file 7: Table S3, Additional file 8: Figure S5, Additional file 10: Figure S6). This may indicate that *A. niger* uses RhaR to (partially) compensate for the loss of XlnR or alternatively that an active XlnR somehow suppresses expression of *rhaR*. A compensation effect between regulators has recently been shown in *A. nidulans* between GalR, XlnR and AraR [39], and previously in *A. niger* for XlnR and AraR [5]. The RG-I main chain is cleaved by endo- (RHG) and exo-rhamnogalacturonase (RGX), unsaturated rhamnogalacturonan hydrolase (URGH), α-rhamnosidase (RHA) and rhamnogalacturonan lyase (RGL), with the assistance of rhamnogalacturonan acetyl esterase (RGAE) [1, 40, 41]. In our study, up-regulation of a number of RG-I degrading-enzymes was observed after 4 h (eight enzymes) and 24 h (thirteen enzymes) of transfer to SBH, correlating well with the up-regulation of RhaR. However, after 48 h of growth the majority of pectinolytic genes involved in RG-I degradation were down-regulated.

Since in CS the amount of L-rhamnose is lower than in SBH, this pathway will not substantially contribute to growth on CS. Indeed, *lraA*, *lraB* and *lraC* were down-regulated after 24 h and 48 h of transfer to CS in the Δ*xlnR* strain. However, *rhaR* was only down-regulated after 48 h of transfer to CS in Δ*xlnR*. In the *xkiA1* mutant, *rhaR* was up-regulated after 4 h of transfer to SBH and down-regulated after 48 h of transfer to CS (Additional file 6: Figure S4). These results correlate with the up-regulation of *lraA*, *lraB,* at all the time points tested, and *lraC* after 4 h and 24 h of transfer to SBH. In the *xkiA1* mutant on CS the results showed up-regulation of *lraA* and *lraC* after 4 h and down-regulation of *lraA*, *lraB* after 24 h and 48 h, and *lraC* after 48 h (Additional file 7: Table S3, Additional file 10: Figure S6). The up-regulation of *rhaR* after 4 h of transfer to SBH might be sufficient to up-regulate the pathway genes at all time points. This up-regulation of *rhaR* correlates with the pectinolytic transcript levels. Five out of seven pectinolytic genes were involved in RG-I degradation and up-regulated after 4 h on SBH in the *xkiA1* mutant. On CS, the down-regulation of *lraA*, *lraB* and *lraC* after 24 h or 48 h, correlated with the down-regulation of the majority of the pectinolytic genes at all the time point.

### D-galacturonic-acid-responsive regulators: GaaR and GaaX

GaaR is a transcription factor required for growth on D-galacturonic acid and for the activation of the D-galacturonic acid responsive genes in *A. niger*. GaaX has been recently described as a repressor, inhibiting the transcription activity of GaaR under non-inducing conditions [42]. The majority of the GaaR-regulated genes encode enzymes needed for the degradation of homogalacturonan (HG), such as exo-polygalacturonases (PGX), endo-polygalacturonases (PGA), pectin methyl esterases (PME) and pectin lyases (PEL) [42]. Also, GaaR is required for induction of D-galacturonic acid reductase (*gaaA*), L-galactonic acid dehydratase (*gaaB*), 2-keto-3-deoxy-L-galactonate aldolase (*gaaC*) and L-glyceraldehyde/L-arabinose reductase (*gaaD*/*larA*) genes involved in D-galacturonic acid catabolism in *A. niger* [43]. After 4 h of transfer to CS or SBH *gaaX* was down-regulated in Δ*xlnR* suggesting that the repression of GaaR by GaaX is removed in the absence of XlnR (Additional file 6: Figure S4). However, after 24 h and 48 h of transfer to SBH and 48 h of transfer to CS *gaaX* was up-regulated in Δ*xlnR*, indicating that removal of repression is only an initial effect in this strain on CS. All genes involved in the D-galacturonic-acid metabolism were down-regulated in both substrates after 4 h of transfer to CS or SBH in Δ*xlnR*. In the *xkiA1* mutant this was only the case for SBH. After 24 h and 48 h of transfer to SBH nearly all D-galacturonic acid pathway genes were up-regulated in both Δ*xlnR* and the *xkiA1* mutant (Additional file 7: Table S3). The exception was *gaaD*/*larA*, which was not differentially expressed in the *xkiA1* mutant. After 24 h on CS *gaaA* and *gaaB* were up-regulated in the *xkiA1* mutant, while *gaaD* was only up-regulated in Δ*xlnR.* After 48 h on CS all genes were down-regulated in both deletion mutants. Expression of *gaaR* was not affected by *xlnR* deletion or *xkiA1* mutation on SBH at all most of the time points tested. However, *gaaR* was down-regulated after 4 h of transfer to CS in Δ*xlnR* and after 24 h in the *xkiA1* mutant (Additional file 9: Table S4). The down-regulation of *gaaR* might be due to other factors at the early time point and not due to a direct effect of *xlnR* deletion in Δ*xlnR* in CS.

The higher content of D-galacturonic acid present in SBH compared to CS likely explains the up-regulation observed after 24 h and 48 h of the first three genes involved in the pathway, while on CS all the pathway genes were down-regulated after 48 h. On SBH, these results correlates with the up-regulation of several HG-degrading enzymes after 24 h and 48 h, while on CS the majority of the genes involved in the HG degradation were down-regulated at all the time points tested, in both deletion mutants.

### The amylolytic regulator AmyR

AmyR is a transcriptional regulator that controls the genes involved in starch degradation, and it has been the first well-studied regulator in several *Aspergillus* species such as *A. nidulans* and *A. oryzae* [44, 45]. Expression of *amyR* was down-regulated at all time points in Δ*xlnR* grown on CS, and after 4 h and 48 h of transfer to CS in the *xkiA1* mutant (Additional file 9: Table S4). These results correlate with the down-regulation of a number of starch degrading-enzymes after 4 and 48 h of transfer to CS in the *xkiA1* mutant (Figs. 4 & 6; Additional file 4: Table S2). After 4 h of transfer to CS in the *xkiA1* mutant nine starch-degrading enzymes were down-regulated: *glaA*, six AGD genes (*agdA*, *agdB*, *agdC*, *agdD*, *agdE* and *agdF*) and two AMY genes (*aamA* and NRRL3_07699). After 48 h of transfer to CS seven starch-degrading enzymes were down-regulated in the *xkiA1* mutant, *glaA* and six AGD genes (*agdA*, *agdB*, *agdC*, *agdD* and *agdE*).

In SBH *amyR* was only down-regulated after 24 h in Δ*xlnR*, and after 4 h in the *xkiA1* mutant. The down-regulation of *amyR* in the *xkiA1* mutant might be part of the initial response of *A. niger* after 4 h of transfer to CS. In Δ*xlnR*, the results did not correlate with the expression of genes encoding starch-degrading enzymes in both substrates, suggesting an indirect effect of XlnR.

### The cellulose regulators ClrA and ClrB

ClrA and ClrB are two TFs involved in the regulation of cellulose degradation, which have been partially characterized in *A. niger* [27]. It was shown that the interaction of two TFs, ClrB and McmA, is necessary for the regulation of *eglA* and *eglB* in *A. nidulans* [46], while in *A. niger*, expression of *cbhA*, *eglC* and *xynA* was shown to be affected by both XlnR and ClrB [45]. Expression of *clrA* was not affected on SBH at any time point tested in either of the deletion mutants. In contrast, *clrB* was down-regulated after 48 h of transfer to SBH in Δ*xlnR*, and up-regulated after 24 h and 48 h of transfer to SBH in the *xkiA1* mutant (Additional file 6: Table S3). In CS, *clrA* was down-regulated at all the time points tested in both deletion mutants, as was *clrB* after 4 h in the *xkiA1* mutant and after 24 h and 48 h in Δ*xlnR*. These results indicate that ClrA or ClrB do not appear to compensate for the absence of XlnR, as observed previously in wheat straw [43]. The role of the homologs of these regulators (Clr1 and Clr2) has been studied in more detail in *Neurospora crassa*, where they are important regulators of genes encoding enzymes that are required for the degradation of cellulose. In contrast, the *N. crassa* XlnR homolog was not necessary for cellulase gene expression or activity [47], demonstrating diverse organization of the regulatory network in fungi. Clr1 and Clr2 appear to be essential in the cellulose degradation in *N. crassa*, but not in *A. niger* where XlnR is the major TF involved in the cellulose and hemicellulose degradation. At this point, no indications for a role of ClrA or ClrB in sugar catabolism have been reported and also our results do not suggest that they affect the expression profiles of the sugar catabolic genes.

## Conclusion

In conclusion, in nature fungi are confronted with mixtures of carbon sources, and therefore likely activate a combination of the gene sets that were observed in response to crude substrates. Our understanding of the hierarchy of the transcriptional regulators and their interaction is still in its infancy, but appears to differ between fungal species. Our results also demonstrate that metabolic and regulatory mutations that result in a similar phenotype on pure sugars can cause significantly different physiology on crude substrates, especially after prolonged exposure. The results of this study confirm that XlnR is the major regulator affecting expression of genes encoding (hemi-)cellulolytic enzymes in *A. niger*, but its influence appears to be dependent on the composition of the available substrates. This composition also strongly affects expression of CAZy genes that are not controlled by XlnR, such as those encoding pectin-degrading enzymes.

Also time influences the expression profiles, in particular during growth on soybean hulls, where the number of differentially expressed genes reduced over time, while the number of differentially expressed genes remained similar on corn stover during the cultivation. This indicates that the dynamic changes in gene expression profiles are strongly substrate dependent.

## Methods

### Strains, media and growth conditions

*A. niger* strains, CBS 141247 (N402, *cspA1*) [48], CBS 141248 (*cspA1,* Δ*argB, nicA1, leuA1,* Δ*xlnR*) [5] and CBS 141251 (N572, *cspA1, xkiA1, nicA1*) [49] were used in our study and were either generated in our laboratory or obtained previously from Dr. J. Visser at Wageningen University. The *A. niger* strains used in this study were grown in minimal (MM) or complete (CM) medium [50] at pH 6.0 and 30 °C with 1.5% of agar. Spore were generates on CM plates containing 2% D-glucose. Liquid cultures of three biological triplicates were inoculated with 10^6^ spores/ml and incubated at 250 rpm and 30 °C in a rotary shaker. Pre-cultures for RNA isolation were performed as described previously [51]. Mycelium was washed with MM and transferred for 4 h, 24 h and 48 h, in 250 ml Erlenmeyer flasks containing 50 ml MM supplemented with 1% CS or 1% SBH for RNA-seq. Mycelium was harvested after 4 h, 24 h and 48 h by vacuum filtration, dried between tissue paper and frozen in liquid nitrogen.

### RNA extraction, cDNA library preparation, RNA-sequencing and RNA data analysis

Total RNA was extracted as described previously [51], while cDNA library preparation and RNA sequencing has also been previously described [52]. Data analysis was performed essentially as in [53]. Filtered reads from each library were aligned to the reference genome (http://genome.jgi.doe.gov/Aspni_NRRL3_1/Aspni_NRRL3_1.home.html) using HISAT version 0.1.4-beta [54], featureCounts [55] was used to generate the raw gene counts using gff3 annotations. On average 98% of the reads were mapped to the genome and 80% of the reads were mapped to a gene. Gene expression was calculated as FPKM (Fragments Per Kilobase of transcript per Million mapped reads). DESeq2 (version 1.10.0) [56] was used to determine which genes were differentially expressed between pairs of conditions. The parameters used to call a gene differently expressed between conditions were adjusted *p*-value <= 0.05 and log2 fold change 0.6 for up-regulated and − 0.6 for down-regulated. Raw gene counts were used for DGE analysis. DESeq2 normalization was based on library size.

PCA was generated using raw counts for all genes obtained from featureCounts [55]. PCA was calculated using the PCA function from FactoMineR package v1.41 [57] keeping 5 dimensions and plotted using ggplot2 v2.2.1 [58] in R statistical language and environment 3.4.0 [59]. Biological replicates are color coded.

RT-qPCR reactions were performed as described previously [51]. The *A. niger* genes studied were: the xylanolytic activator (*xlnR*), endoxylanase (*xynB*), β-xylosidase (*xlnD*), α-glucuronidase (*aguA*), rhamnogalacturonan lyase B (*rglB*), exorhamnogalacturonase A (*rgxA*) and rhamnogalacturonan acetyl esterase A (*rgaeA*). Histone gene (H2S) was used as reference gene. The sequences of all primers for RT-qPCR analysis were designed using the Primer Express 3.0 software (Applied Biosystems, Foster City, CA, USA) and their optimal primer concentrations and efficiency have been previously described [51, 60]. Three biological and three technical replicates were analyzed.

## Supplementary information

**Additional file 1: Figure S1.** Principle Component Analysis (PCA) demonstrating the high reproducibility of the biological replicates.

**Additional file 2: Figure S2.** GO-term enrichment analysis of differentially expressed genes.

**Additional file 3: Table S1.** GO-term enrichment analysis of differentially expressed genes.

**Additional file 4: Table S2.** Expression of selected CAZymes involved in the degradation of plant biomass in *A. niger*. The comparisons between strains are Δ*xlnR* over the wild-type and *xkiA1* over the wild-type. The cut-off for differential expression is log2 fold change > 0.6 (cells marked red if up-regulated) and log2 fold change <0.6 (cells marked green if down-regulated) and adjusted *p*-value <=0.05(cells marked yellow).

**Additional file 5: Figure S3.** Heatmap reflecting the differential expression of CAZYme-encoding genes The polysaccharides the genes are related to are indicated in the grid behind the heat map.

**Additional file 6: Figure S4.** Comparison of RNAseq and Q-PCR expression profiles of seven selected genes. *aguA* = alpha-glucuronidase (NRRL3_01069), *xlnD* = beta-xylosidase (NRRL3_02451), *xynB* = endoxylanase (NRRL3_01648), *xlnR* = (hemi-)cellulolytic transcriptional activator, *rglB* = rhamnogalacturonan lyase (NRRL3_10115), *rgxA* = exorhamnogalacturonase (NRRL3_02832), *rgaeA* = rhamnogalacturonan acetyl esterase (NRRL3_00169). Graphs depict the log2 of the fold change of the averaged expression values of the wild type vs the indicated mutant.

**Additional file 7: Table S3.** Expression of known genes involved in central carbon metabolism in *A. niger*. The comparisons between strains are Δ*xlnR* over the wild-type and *xkiA1* over the wild-type. The cut-off for differential expression is log2 fold change > 0.6 (cells marked red if up-regulated) and log2 fold change < 0.6 (cells marked green if down-regulated) and adjusted p-value < 0.05(cells marked yellow).

**Additional file 8: Figure S5.** Representation of pentose catabolic pathway, including expression profiles of the genes involved in the pathway.

**Additional file 9: Table S4.** Expression of known regulators genes involved in plant biomass degradation in *A. niger*. The comparisons are between deletion mutants over the wild-type. The cutoff for differential expression if up-regulated log2 fold-change > 0.6 (cells marked dark grey) and log2 fold-change <− 0.6 if down-regulated (cells marked light grey) and adjusted p-value <=0.05 (*).

**Additional file 10: Figure S6.** Representation of L-rhamnose pathway, including expression profiles of the genes involved in the pathway.

## Data Availability

The RNA-seq data have been deposited at the Sequence Read Archive at NCBI with individual sample BioProject Accession Numbers SRP112071 (https://www.ncbi.nlm.nih.gov/sra/?term=srp112071), SRP112127 (https://www.ncbi.nlm.nih.gov/sra/?term=SRP112127) and SRP112158 (https://www.ncbi.nlm.nih.gov/sra/?term=SRP112158).

## Abbreviations

CM: complete medium
CS: corn stover
MM: minimal medium
PCP: pentose catabolic pathway
PPP: pentose phosphate pathway
SBH: soy bean hulls
XkiA: D-xylulokinase
XlnR: (hemi-)cellulolytic transcriptional activator
