# Supplementary figures and images for: Transcriptome analysis of Aspergillus niger xlnR and xkiA mutants grown on corn Stover and soybean hulls reveals a highly complex regulatory network
Source: BMC Genomics. 2019 Nov 14;20:853. doi: 10.1186/s12864-019-6235-7 (PMC6854810; doi:10.1186/s12864-019-6235-7)

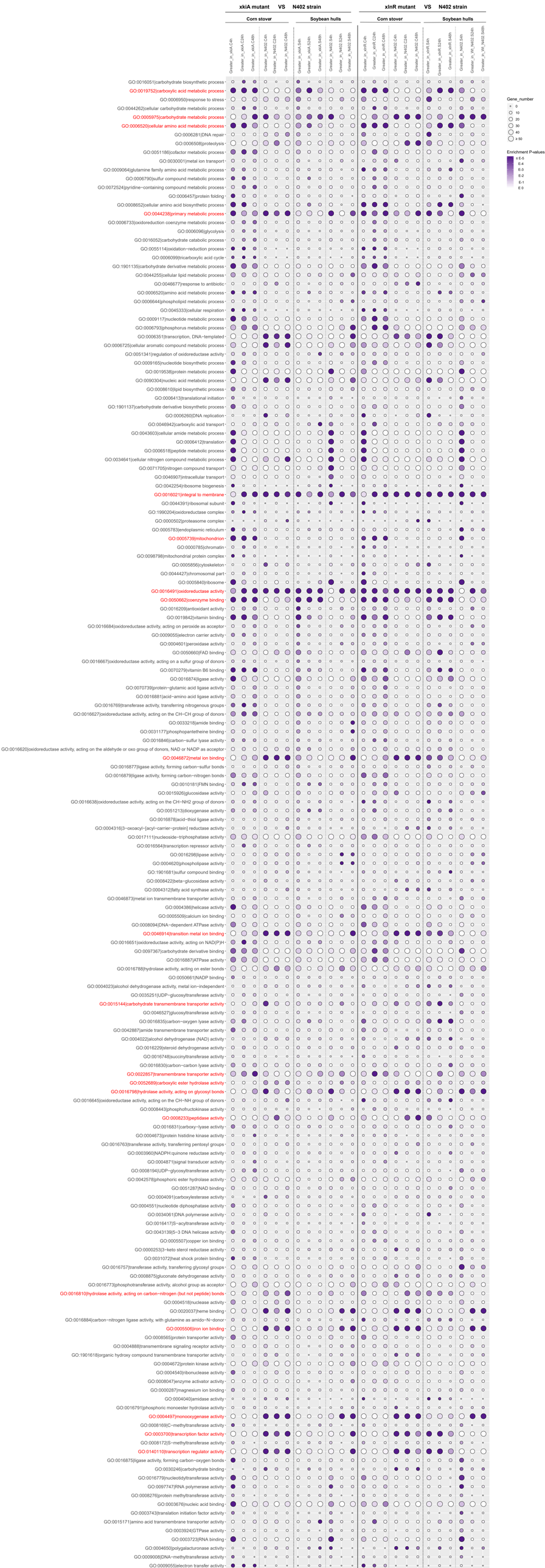

Supplement: Supplementary file 2 — Additional file 2: Figure S2. GO-term enrichment analysis of differentially expressed genes. [file 12864_2019_6235_MOESM2_ESM.zip › Additional File 2.pdf]

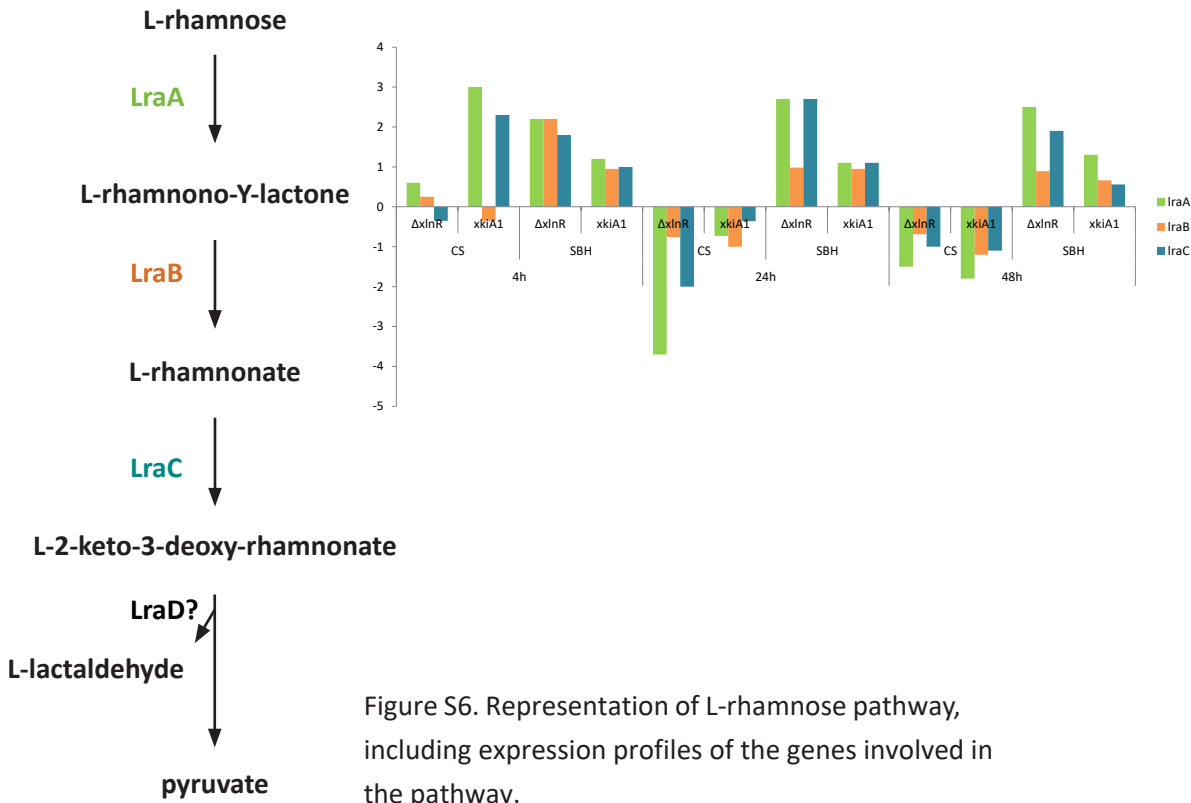

Supplement: Supplementary file 10 — Additional file 10: Figure S6. Representation of L-rhamnose pathway, including expression profiles of the genes involved in the pathway. [file 12864_2019_6235_MOESM10_ESM.pdf]
