# Supplementary material for: Transcriptome analysis of Aspergillus niger xlnR and xkiA mutants grown on corn Stover and soybean hulls reveals a highly complex regulatory network
Source: BMC Genomics. 2019 Nov 14;20:853. doi: 10.1186/s12864-019-6235-7 (PMC6854810; doi:10.1186/s12864-019-6235-7)
Supplement: Supplementary file 5 — Additional file 5: Figure S3. Heatmap reflecting the differential expression of CAZYme-encoding genes The polysaccharides the genes are related to are indicated in the grid behind the heat map. [file 12864_2019_6235_MOESM5_ESM.zip › Figure S3.pdf]

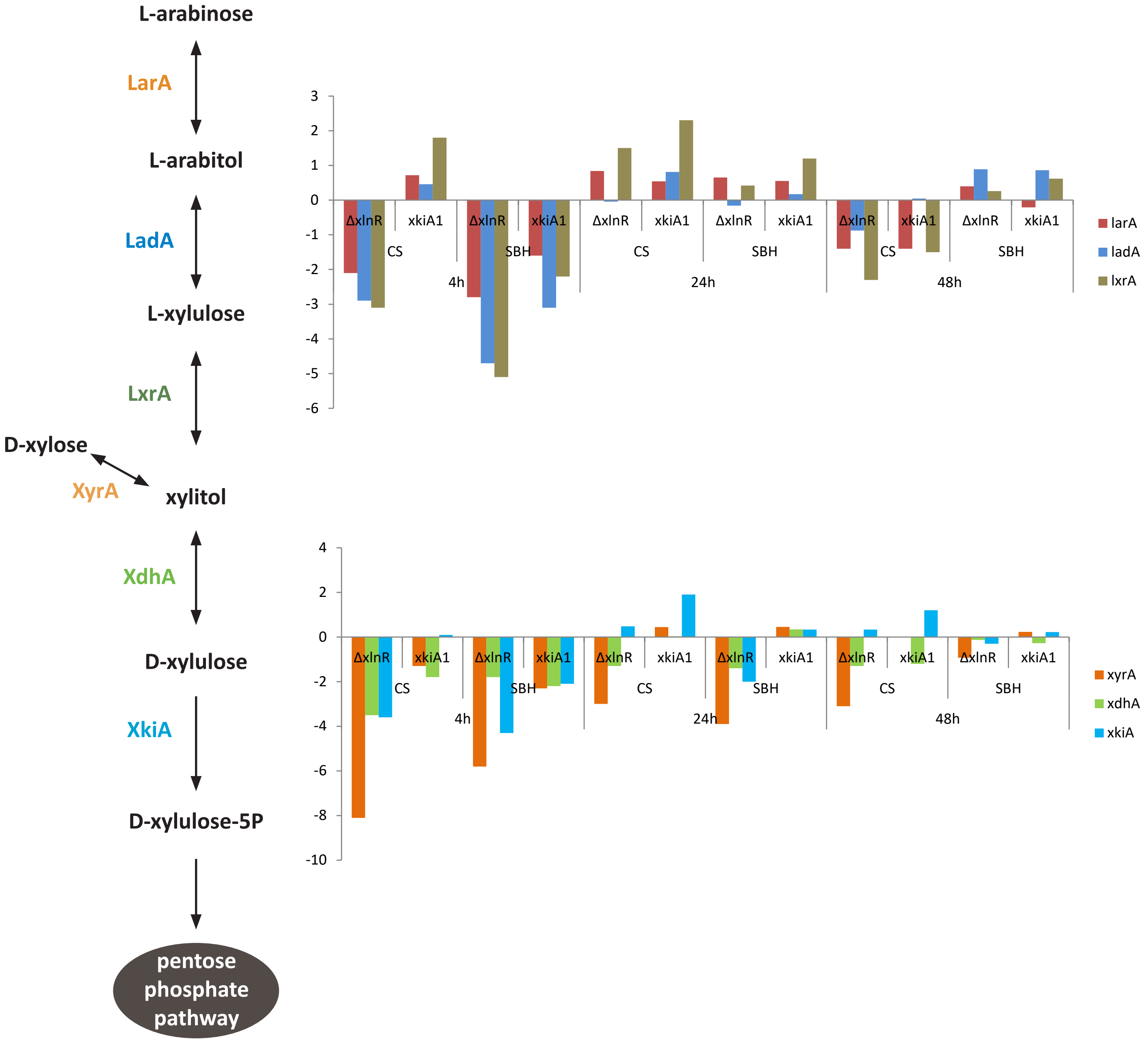

Figure S3. Representation of pentose catabolic pathway, including expression profiles of the genes involved in the pathway.
