# Supplementary material for: Transcriptome analysis of Aspergillus niger xlnR and xkiA mutants grown on corn Stover and soybean hulls reveals a highly complex regulatory network
Source: BMC Genomics. 2019 Nov 14;20:853. doi: 10.1186/s12864-019-6235-7 (PMC6854810; doi:10.1186/s12864-019-6235-7)
Supplement: Supplementary file 6 — Additional file 6: Figure S4. Comparison of RNAseq and Q-PCR expression profiles of seven selected genes. aguA = alpha-glucuronidase (NRRL3_01069), xlnD = beta-xylosidase (NRRL3_02451), xynB = endoxylanase (NRRL3_01648), xlnR = (hemi-)cellulolytic transcriptional activator, rglB = rhamnogalacturonan lyase (NRRL3_10115), rgxA = exorhamnogalacturonase (NRRL3_02832), rgaeA = rhamnogalacturonan acetyl esterase (NRRL3_00169). Graphs depict the log2 of the fold change of the averaged expression values of the wild type vs the indicated mutant. [file 12864_2019_6235_MOESM6_ESM.zip › Additional File 6.pdf]

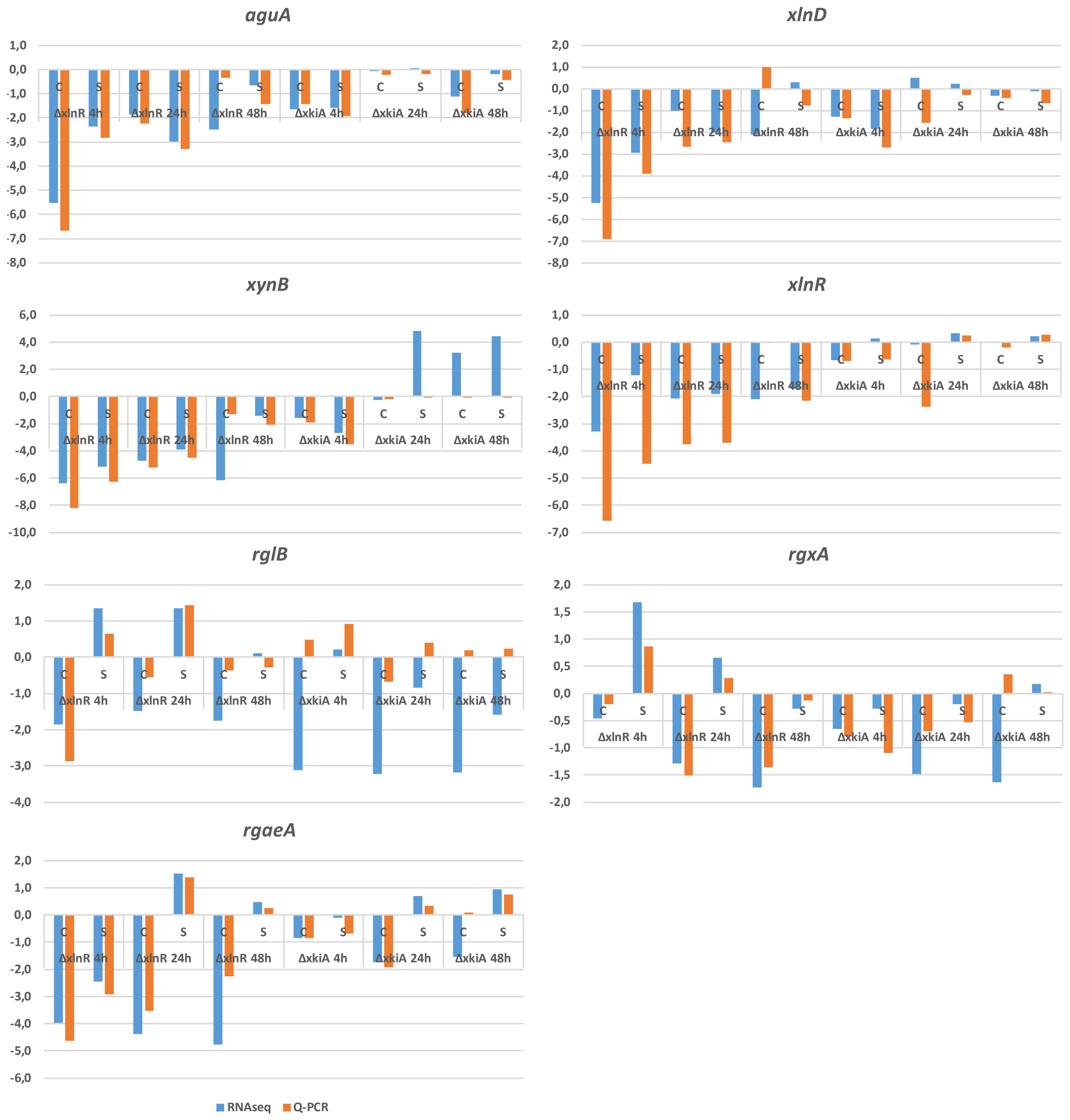

Figure S4. Comparison of RNAseq and Q-PCR expression profiles of seven selected genes. *aguA* = alpha-glucuronidase (NRRL3_01069), *xlnD* = beta-xylosidase (NRRL3_02451), *xynB* = endoxylanase (NRRL3_01648), *xlnR* = (hemi-)cellulolytic transcriptional activator, *rglB* = rhamno-galacturonan lyase (NRRL3_10115), *rgxA* = exorhamnogalacturonase (NRRL3_02832), *rgaeA* = rhamnogalacturonan acetyl esterase (NRRL3_00169). Graphs depict the log2 of the fold change of the averaged expression values of the wild type vs the indicated mutant.
