# Supplementary material for: Transcriptome analysis of Aspergillus niger xlnR and xkiA mutants grown on corn Stover and soybean hulls reveals a highly complex regulatory network
Source: BMC Genomics. 2019 Nov 14;20:853. doi: 10.1186/s12864-019-6235-7 (PMC6854810; doi:10.1186/s12864-019-6235-7)
Supplement: Supplementary file 8 — Additional file 8: Figure S5. Representation of pentose catabolic pathway, including expression profiles of the genes involved in the pathway. [file 12864_2019_6235_MOESM8_ESM.pdf]

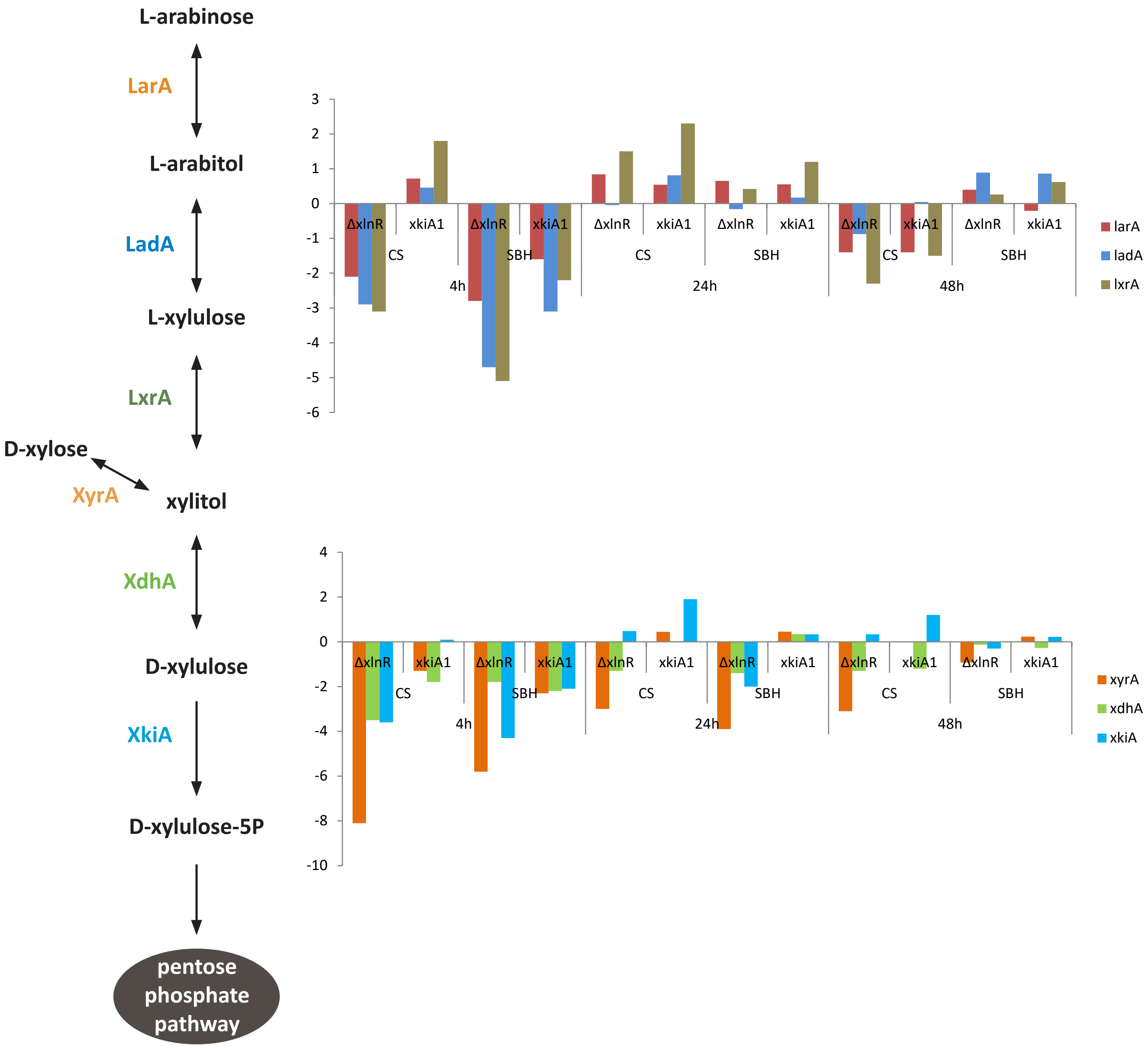

Figure S5. Representation of pentose catabolic pathway, including expression profiles of the genes involved in the pathway.
