# Supplementary material for: Transcriptome analysis of Aspergillus niger xlnR and xkiA mutants grown on corn Stover and soybean hulls reveals a highly complex regulatory network
Source: BMC Genomics. 2019 Nov 14;20:853. doi: 10.1186/s12864-019-6235-7 (PMC6854810; doi:10.1186/s12864-019-6235-7)
Supplement: Supplementary file 1 — Additional file 1: Figure S1. Principle Component Analysis (PCA) demonstrating the high reproducibility of the biological replicates. [file Figure S1.pdf › Figure S1.pdf]

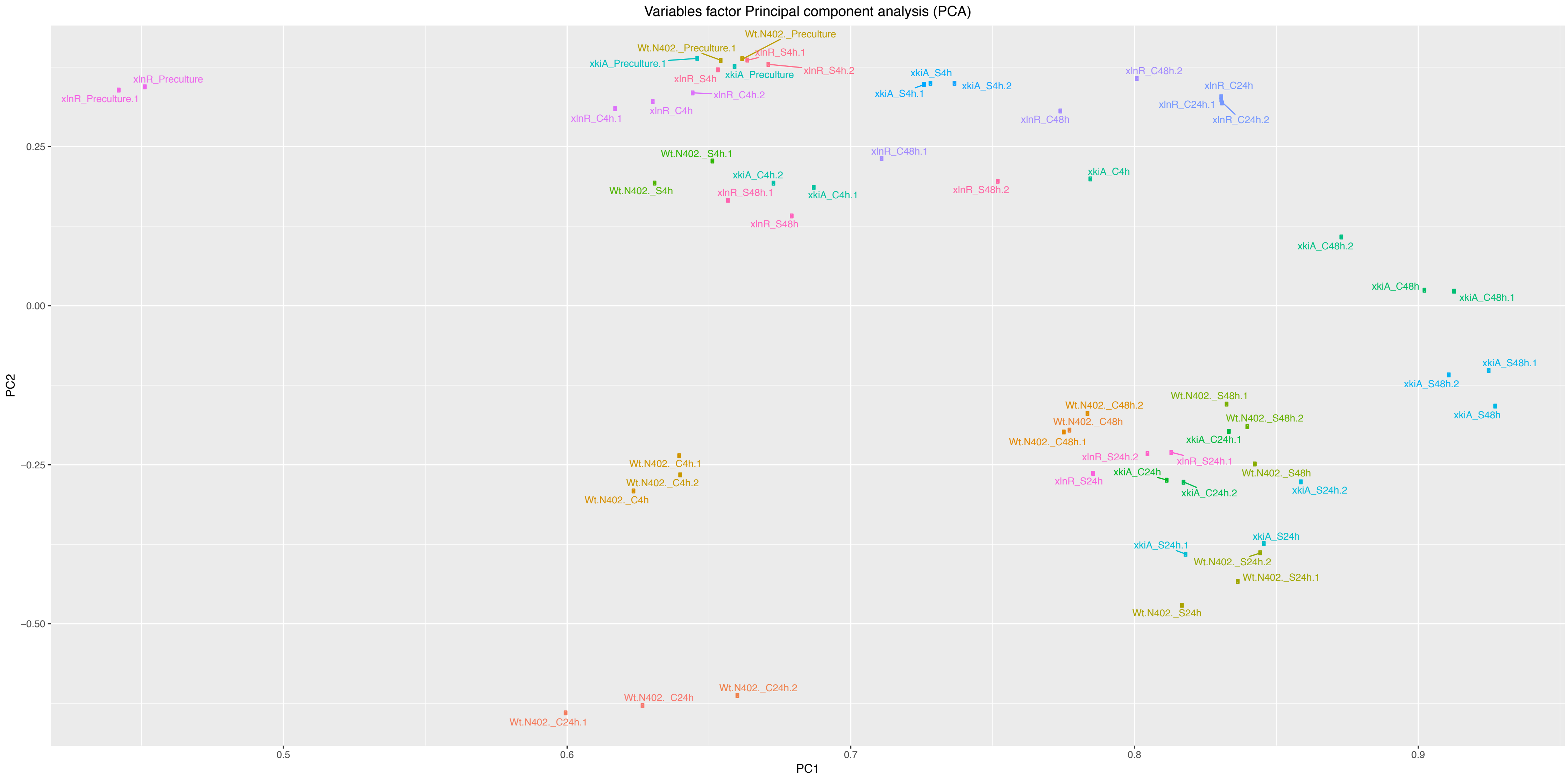

Figure S1. Principle Component Analysis (PCA) demonstrating the high reproducibility of the biological replicates.
